# Impact of Maternal Obesity on the Metabolism and Bioavailability of Polyunsaturated Fatty Acids during Pregnancy and Breastfeeding

**DOI:** 10.3390/nu13010019

**Published:** 2020-12-23

**Authors:** Daniela Álvarez, Yasna Muñoz, Macarena Ortiz, Manuel Maliqueo, Raphaël Chouinard-Watkins, Rodrigo Valenzuela

**Affiliations:** 1 Endocrinology and Metabolism Laboratory, West Division, Faculty of Medicine, University of Chile, Santiago 8380000, Chile; danialvareza@gmail.com (D.Á.); yasnamunoz.nut@gmail.com (Y.M.); macapaz.ortiz@gmail.com (M.O.); mmaliqueo@med.uchile.cl (M.M.); 2 Department of Nutritional Sciences, Faculty of Medicine, University of Toronto, Toronto, ON M5S1A8, Canada; raphael.chouinard.watkins@utoronto.ca; 3 Nutrition Department, Faculty of Medicine, University of Chile, Santiago 8380000, Chile

**Keywords:** fatty acids, obesity, pregnancy, breastfeeding, fatty acid metabolism, fatty acid bioavailability

## Abstract

Prenatal and postnatal development are closely related to healthy maternal conditions that allow for the provision of all nutritional requirements to the offspring. In this regard, an appropriate supply of fatty acids (FA), mainly *n*-3 and *n*-6 long-chain polyunsaturated fatty acids (LCPUFA), is crucial to ensure a normal development, because they are an integral part of cell membranes and participate in the synthesis of bioactive molecules that regulate multiple signaling pathways. On the other hand, maternal obesity and excessive gestational weight gain affect FA supply to the fetus and neonate, altering placental nutrient transfer, as well as the production and composition of breast milk during lactation. In this regard, maternal obesity modifies FA profile, resulting in low *n*-3 and elevated *n*-6 PUFA levels in maternal and fetal circulation during pregnancy, as well as in breast milk during lactation. These modifications are associated with a pro-inflammatory state and oxidative stress with short and long-term consequences in different organs of the fetus and neonate, including in the liver, brain, skeletal muscle, and adipose tissue. Altogether, these changes confer to the offspring a higher risk of developing obesity and its complications, as well as neuropsychiatric disorders, asthma, and cancer. Considering the consequences of an abnormal FA supply to offspring induced by maternal obesity, we aimed to review the effects of obesity on the metabolism and bioavailability of FA during pregnancy and breastfeeding, with an emphasis on LCPUFA homeostasis.

## 1. Introduction

**Figure 1:**
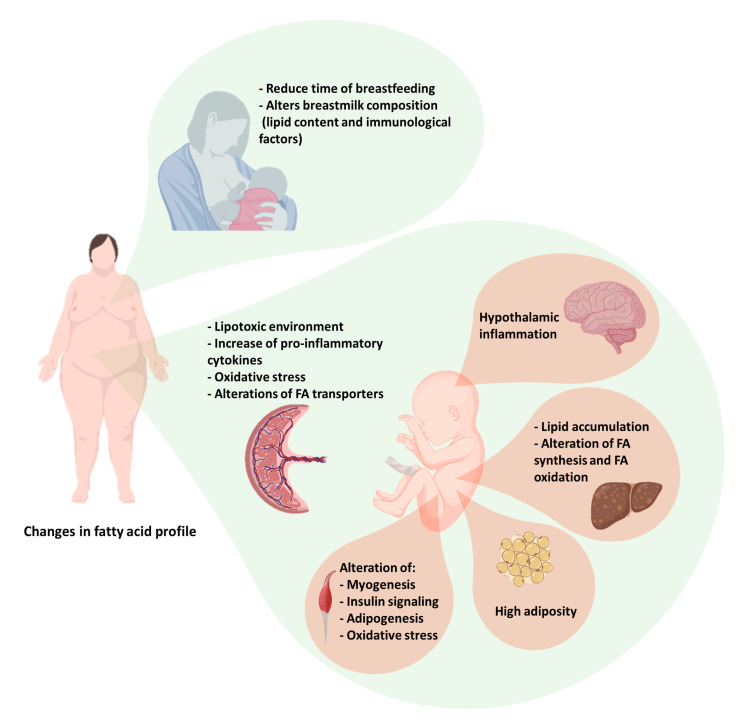
Maternal obesity is associated with changes in circulating fatty acid (FA) profile and placental alterations including a lipotoxic environment, a pro-inflammatory state, and elevated oxidative stress, that altogether lead to modifications in FA transporters that affect the development of fetal organs such as the brain, liver, adipose tissue, and skeletal muscle. Moreover, maternal obesity reduces the time of breastfeeding and alters the FA composition of breast milk.

Maternal obesity and excessive gestational weight gain (GWG) are frequently associated with alterations in carbohydrate and lipid metabolism, abnormal levels of pregnancy hormones, and a pro-inflammatory state. These disturbances increase the risk of maternal and fetal complications such as gestational diabetes mellitus, hypertensive disorders of pregnancy, cesarean delivery, lung disease, miscarriage, still birth, fetal chromosomic anomalies, preterm birth, and fetal macrosomia [1,2]. Moreover, adiposity excess alters the placental nutrient transfer and modifies the composition of breast milk, affecting the development and genetic programing of multiple fetal organs (liver, adipose tissue, skeletal muscle, and brain, among others) (Figure 1). Altogether, these changes confer to the offspring a higher risk of obesity and related complications, as well as of neuropsychiatric disorders, asthma, and cancer [3,4,5,6,7].

During pregnancy and lactation, all the nutritional requirements necessary to ensure an appropriate growth of the fetus and neonate are transferred from the mother [8]. Fetal development is intimately linked to the placental exchange function, such that newborn size reflects the net nutrient transfer through the placenta [9]. Likewise, breast milk provides fats, proteins, carbohydrates, immune cells, and bioactive molecules for optimal infant growth [10,11,12].

Fatty acids (FA) participate in fat mass accretion, regulation of metabolic pathways, and visual and brain development. Depending on the number of double bounds, FA can be classified as saturated (SFA), monounsaturated (MUFA), or polyunsaturated (PUFA) [13]. Of these, *n*-3 and *n*-6 long chain PUFA (LCPUFA) are of particular interest because they are an integral part of cell membranes and participate in the synthesis of bioactive molecules that regulate multiple signaling pathways [13]. The supply of essential FA to the fetus will depend entirely on maternal consumption, placental transport and metabolism of FA [14]. Therefore, a balanced diet for pregnant and lactating women must include an appropriate supply of FA. In humans, the synthesis of *n*-3 and *n*-6 PUFA requires α–linolenic acid (C18:3*n*-3, ALA) and linoleic acid (C18:2*n*-6, LA), respectively. These FA are considered essential and cannot be synthesized in mammals due to a lack of the enzyme delta-12 desaturase (FADS2) and delta-15 desaturase (FADS3) [15]. ALA and LA are desaturated by desaturases (FADS1 or Δ-5 and FADS2 or Δ-6) and elongated by elongases (ELOVL 2 and 5) to produce, notably, docosahexaenoic acid (C22:6*n*-3, DHA) or arachidonic acid (C20:4*n*-6, AA). AA and DHA are crucial for the formation and maturation of the brain and other organs during fetal and neonatal development. These characteristics emphasize the importance of an adequate intake of LCPUFA during these periods. Some of the LCPUFA elongation products can also be oxidized through peroxisomal β-oxidation [16].

In general, obesity is characterized by high energy intake due to excessive consumption of food rich in refined carbohydrates and saturated fats, and low consumption of sea foods, vegetables, fruits, and legumes (important sources of dietary fiber and natural antioxidants) [17,18,19]. This dietary pattern is associated with an imbalance in FA intake which is reflected by an excessive consumption of *n*-6 PUFA and low *n*-3 PUFA consumption [19]. In this regard, the main sources of DHA are fatty fish and seaweed [20]. Moreover, measurements using traceable carbon 13 (13C) FA stable isotopes and the quantification of the gene and protein expression of FA transporters in the placenta indicate that obesity affects the transplacental transport of FA that are necessary for fetal development, among which DHA is one of the most important [21]. Breast milk adjusts to neonatal demand by varying the composition of bioactive compounds and nutrients, such as FA, during lactation [22].

The short- and long-term consequences of maternal obesity are important public health issues. For this reason, extensive research around the world has been focused on the role of LCPUFA in fetal and neonatal growth, with the purpose of improving nutritional quality during these periods. In view of these considerations, the aim of this review was to summarize the available information about the impact of obesity on the metabolism and bioavailability of PUFA during pregnancy and breastfeeding.

## 2. Material and Methods

This review included both in vivo and in vitro studies aimed at evaluating the effect of obesity on the metabolism and bioavailability of PUFA during pregnancy and breastfeeding. Searches were performed using the PubMed database from the National Library of Medicine—National Institutes of Health. The following keywords were used for the literature search: maternal obesity and PUFA metabolism and fetal development; maternal obesity and PUFA metabolism and breastfeeding; maternal obesity and PUFA metabolism and placenta.

## 3. Maternal Obesity and Its Consequences for the Offspring

Maternal pre-pregnancy obesity is independently associated with fetal overgrowth and with total body adiposity, abdominal fat accumulation, and lower fat free-mass in neonates [23,24,25,26,27,28]. These features are aggravated by an excessive GWG during mid and late pregnancy at the time of higher fat accretion in the fetus [25,29,30]. Moreover, high adiposity in neonates is related to insulin resistance, hyperinsulinemia, and a pro-inflammatory status characterized by high circulating levels of leptin and interleukin-6 (IL-6) [31,32]. In addition to these features, the intrahepatic fat content of neonates during the first week after birth is correlated with maternal body mass index (BMI) in obese women with gestational diabetes [33,34].

The effects of maternal obesity on offspring are maintained during childhood and even adulthood, such that it has been reported that obese women at the highest range of GWG confer a high risk of obesity to their offspring at 5 years old, and this higher risk is maintained during adolescence [35]. Moreover, maternal overweight during early pregnancy increases the risk factors for metabolic and cardiovascular diseases in adolescents regardless of their current BMI [36]. Interestingly, in a large study based on a Swedish nationwide register, the authors showed that the hazard risk for cardiovascular disease in offspring aged between 1 and 25 years old born to obese pregnant women ranged from 1.10 (95% CI 0.97–1.25) for overweight to 2.51 (95% CI 1.60–3.92) for obesity grade III [37]. In this regard, mice born to high fat diet (HFD)-induced obese dams had hyperphagia, physical inactivity, and altered adipocyte metabolism, leading to high adiposity and obesity. These results suggest that the metabolic changes associated with maternal obesity are programmed during prenatal life and maintained throughout the lifespan [38].

It is important to note that excessive GWG during pregnancy is a determinant factor on the offspring’s risk of developing metabolic disorders. Observations from the Generation R study conducted in the city of Rotterdam in the Netherlands showed that a higher weight gain in early, but not in mid or late pregnancy, was associated with increased risks of childhood overweight and clustering of cardio-metabolic factors at 6 years old [39]. Moreover, the effects of maternal pre-pregnancy BMI on childhood BMI, waist circumference, subcutaneous adipose tissue, and HDL-cholesterol at 10 years of age were attenuated by a reduced weight gain during pregnancy, which was independent of the current diet and physical activity of the child [40]. Regarding hepatic fat accumulation, adolescents born to mothers with a BMI over 30 kg/m^2^ before pregnancy had an increased hepatic fat fraction independent of gestational diabetes mellitus (GDM) and children adiposity [41]. Together with these metabolic and cardiovascular risk factors, it has been reported that children and adolescents born to mothers with obesity and/or excessive GWG present poor neurodevelopmental outcomes, with greater risk of attention deficit-hyperactivity disorder, autism spectrum disorder (ASD), developmental delay, and emotional-behavioral problems [42]. Furthermore, excessive weight and obesity during pregnancy have been associated with increased rates of malformations of the nervous system and epilepsy, which were worsened by the presence of neonatal hypoglycemia, respiratory distress syndrome, and neonatal jaundice [43].

Interestingly, the risk of developing overweight in children from 2 to 14 years of age born to women with maternal pre-pregnancy obesity was aggravated by the lack of breast-feeding with an OR of 6.1 (95% CI 2.9–13.1) when the two factors were present [44]. Similarly, a mice model of gestational and lactation overnutrition showed that the offspring had a severe metabolic phenotype in adulthood when they were exposed to HFD, and this was characterized by an increase in weight gain and adiposity, in addition to glucose homeostasis disturbances and lipid accumulation in the liver [45].

Some studies indicate that the offspring risk of developing metabolic, cardiovascular, and mental disorders differs between males and females. In this regard, at the neonatal period only, girls born to mothers with obesity exhibited increased skinfold thickness and serum leptin concentrations [46]. On the other hand, another study showed that at ages between 2 and 6 years old, boys born to mothers with obesity have higher body fat compared to those born to normal and overweight mothers, whereas no differences were observed in girls [47]. However, in adult women, the risk of developing type 2 diabetes increased according to the BMI of their mother during pregnancy [48]. The development of non-alcoholic fatty liver disease (NAFLD) was also associated with maternal obesity and higher GWG at early-mid pregnancy only in female offspring [49]. On the other hand, boys whose mothers were severely obese have shown more behavioral problems than those born to normal-weight mothers [50]. One study showed that adequate breastfeeding protects against adiposity excess only in boys born to overweight mothers [51], while another study showed that the clustering of cardiometabolic risk factors associated with maternal obesity is not influenced by breastfeeding [52]. In summary, maternal obesity during pregnancy and breastfeeding is associated with metabolic, cardiovascular, and behavioral effects in the offspring which might be sex-dependent, although the effects and consequences are highly heterogenous and not completely clear between males and females. Further studies are needed to clarify these issues.

## 4. Effects of Obesity on FA Metabolism during Pregnancy

### 4.1. Fatty Acid during Pregnancy

Maternal body fat accumulation during early pregnancy allows for the storage of important amounts of FA, especially LCPUFA derived from diet and maternal metabolism [53]. During the last trimester of gestation, the accumulation of fat depots in maternal tissues declines because of higher lipolysis and mobilization of triacylglycerols (TAG). The elevations of serum TAG, very low-density lipoprotein (VLDL), and non-esterified fatty acids (NEFA) increase FA bioavailability to be transferred to the fetus [53,54,55,56]. These metabolic changes are a consequence of pregnancy and associated with modifications in the synthesis and secretion of human placental lactogen, placenta growth hormone, cortisol, progesterone, estrogens, and adipokines derived from adipose tissue [57]. In this regard, in pregnant rats, estradiol levels are strongly correlated with the mRNA expression of *Fads2* mainly during the mid-pregnancy, whereas serum progesterone concentrations are associated with the hepatic content of LCPUFA intermediates, particularly *n*-6 docosapentaenoic acid (C22:5*n*-6, DPA *n*-6) [58]. Taken together, these results suggest a placental control in maternal LCPUFA metabolism. In the maternal bloodstream, FA are contained in different proportions in TAG, phospholipids, and cholesterol esters or circulate as NEFA [56]. The FA composition in maternal erythrocytes indicates that the metabolism of SFA, MUFA, and PUFA is modified throughout pregnancy, showing an increase in palmitoleic acid (C16:1*n*-7, POA), nervonic acid (C24:1*n*-9, NA), *n*-3 LCPUFA such as n-3 docosapentaenoic acid (C22:5*n*-3, DPA *n*-3) and DHA, and *n*-6 LCPUFA such as LA and AA during pregnancy [59,60]. On the other hand, eicosapentaenoic acid (C20:5*n*-3, EPA) levels do not change significantly during gestation in erythrocytes or plasma. Interestingly, despite the increase in the absolute FA concentration during pregnancy, the ratio of essential PUFA in erythrocytes such as LA and ALA on total FA decreases, suggesting that pregnancy is associated with a reduction in their relative amounts in maternal circulation, probably due to the high transfer of these PUFA to the fetus [61]. In the serum, most of the palmitic acid (C16:0, PA) and oleic acid (C18:1*n*-9, OA) are found in TAG, whereas LA and DHA are found in phospholipids and TAG. This differential serum distribution between FA may influence their uptake and transfer [62]. Elegant in vivo studies that track FA metabolism with stable isotope FA tracers administered orally to pregnant women 4 to 12-h before labor have demonstrated a preferential transfer of DHA over other FA, such as PA, OA, and ALA [62,63]. In general, this selective enrichment of LCPUFA, mainly DHA and AA, in the fetal circulation during pregnancy and lactation is known as biomagnification [61,64]. These findings have given rise to the hypothesis that DHA and AA have to be transferred preferentially across the placenta to support their rapid accretion in the fetal nervous tissue during the period of brain growth spurt [65].

The dietary FA recommendations during pregnancy are shown in Table 1. According to the committee of the United Nations Food and Agriculture Organization and the World Health Organization (WHO), PUFA intake during pregnancy and lactation should represent between 20 and 35% of the total fat intake [66]. The main emphasis must be on dietary DHA and AA requirements because the risk of deficiency of these PUFA increases during pregnancy due to fetal neurodevelopment requirements [67]. Therefore, several organizations including the Food and Agriculture Organization, the WHO, the Perinatal Lipid Nutrition Project (PeriLip), and the Early Nutrition Project (EARNEST) of the European Commission recommend a daily intake of at least 300 mg of EPA and 200 mg of DHA per day or the consumption of one to two portions of fatty fish per week both in pregnant and lactating women [66,68]. Moreover, it has been indicated that the daily intake of AA should be around 800 mg [66]. The Food and Nutrition Board of the Institute of Medicine recommends a daily intake of LA and ALA during pregnancy at around 13 g per day and 1.4 g per day, respectively [69], although there is no clear consensus on the dietary requirements of these FA during pregnancy.

The beneficial effects of DHA during pregnancy or early postnatal period have been widely documented, showing a reduction in the risk of preeclampsia, preterm birth, and depression in the mothers, in addition to increases in birthweight and improvements in brain and visual development [72,73]. In support of these findings, a study by Much et al. on healthy pregnant women that received a fish oil supplement with 1200 mg *n*-3 LCPUFA (1020 mg DHA and 180 mg EPA), starting at week 14 of gestation and until the end of lactation, showed that maternal erythrocyte concentrations of *n*-3 LCPUFA at week 32 of gestation were positively associated with the children weight and length at birth [74]. On the other hand, AA and *n*-6 LCPUFA have been negatively correlated with the BMI and ponderal index at one year of life [74]. A randomized double-blind clinical trial conducted in 144 pregnant women found that infants of mothers who received 200 mg of DHA during pregnancy had a lower BMI at 21 months of age but no difference at 6 years old, suggesting that the effects of maternal DHA disappear as children grow older [75,76].

Pregnant women with overweight or obesity have an altered lipid profile characterized by elevated TAG, total cholesterol, LDL-cholesterol, and VLDL cholesterol, but lower HDL-cholesterol in comparison to women with normal weight [77,78,79,80]. In a small sample of participants, Scifres et al. observed that maternal serum levels of AA, both at the first and late second trimester of gestation, are higher in overweight/obese women in comparison to normal-weight women [60]. On the other hand, a large cohort study involving 5636 women at mid pregnancy described high SFA and low *n*-3 PUFA serum levels in women with pre-pregnancy obesity. Excessive GWG increases the serum levels of SFA, MUFA, and *n*-6 PUFA such as dihomo-γ-linolenic acid (C20:3*n*-6, DGLA) and AA, but decreases LA [81]. However, the FA profile in red blood cells showed lower levels of MUFA and LA but higher levels of DHA in women with maternal obesity [82]. Interestingly, a metabolomic analysis showed that the serum levels of NEFA *n*-6 PUFA and phosphatidylcholines containing DGLA were positively correlated with pre-pregnancy BMI [83].

**Figure 2:**
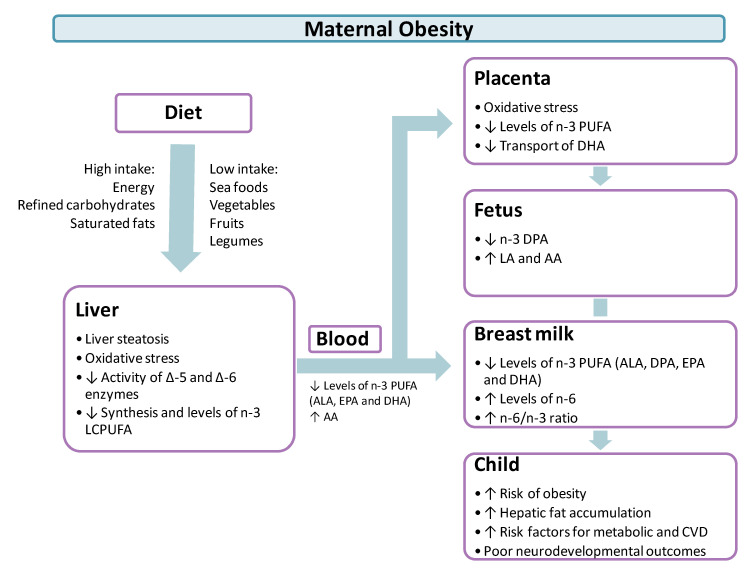
Impact of maternal obesity on the metabolism of *n*-3 and *n*-6 LCPUFA during pregnancy and lactation. An unhealthy diet, commonly associated with obesity, along with modifications in FA metabolism in the maternal liver, leads to low circulating levels of *n*-3 PUFA and elevated circulating levels of *n*-6 PUFA. These modifications are also present in the placenta, fetus, breast milk, and, consequently, in the child. LCPUFA, long chain polyunsaturated fatty acid; PUFA, polyunsaturated fatty acid; ALA, α–linolenic acid; AA, arachidonic acid; DHA, docosahexaenoic acid; DPA, docosapentaenoic acid; EPA, eicosapentaenoic acid; *n*-6/*n*-3 ratio, total polyunsaturated fatty acid *n*-6/*n*-3 ratio; CVD, cardiovascular disease; Delta -5 (Δ-5) desaturase; Delta-6 (Δ-6) desaturase.

Although changes in the blood FA profile seem to be determined mainly by maternal diet, obesity during gestation can also provoke modifications in PUFA metabolism in the maternal liver, affecting PUFA circulating levels (Figure 2). For instance, women with gestational obesity have a high prevalence of NAFLD, which might be associated with a reduction in liver PUFA synthesis due to a reduction in the activity of FADS1 and FADS2 as a result of an abnormal fat accumulation and oxidative stress [84,85,86,87]. The latter is a product of an elevated production of reactive oxidant molecules that exceeds the capacity of the cell’s antioxidant defense mechanisms [88]. Pregnancy is characterized by a state of oxidative stress that increases progressively from the late first trimester onward [89] and gestational obesity seems to aggravate this condition. For example, in obese rats there is an increase of reactive oxidative species (ROS) in the maternal liver compared to normal weight rats [90]. Importantly, according to the antioxidant and anti-inflammatory properties of *n*-3 PUFA in adults, it has been suggested that the maternal consumption of these FA could limit oxidative damage during pregnancy [91].

### 4.2. Placental Fatty Acid Transport

The placenta is responsible for the maternal-fetal transfer of oxygen, carbon dioxide, water, and all the nutrients necessary for the development of the fetus [92]. The exchange function occurs in placental chorionic villi that are composed of the syncytiotrophoblast, a multinucleated and polarized epithelium that comes from the fusion of mononuclear trophoblasts and is considered the transporter epithelium of the placenta [93]. The apical membrane has microvilli that are in contact with the maternal blood, and the basal membrane is in contact with the fetal capillaries. Because only very small substrates can cross both membranes of the syncytiotrophoblast, macro and micronutrients are transported through nutrient transporters that are highly expressed in this epithelium [94].

**Figure 3:**
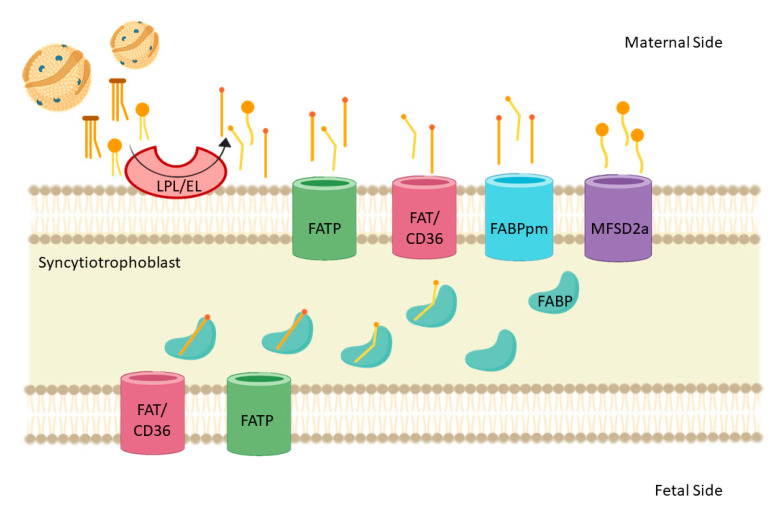
Schematic representation of placental fatty acid transport. Maternal triglycerides are hydrolyzed to non-esterified fatty acid (NEFA) by placental lipases (lipoprotein lipase (LPL) and endothelial lipoprotein lipase 9 (EL9). Then, the placenta can uptake and move the NEFA across the membrane through a flip-flop mechanism or with the help of binding proteins and transporters. FABP: fatty acid binding protein; FAT/CD36: fatty acid translocase; FATP: fatty acid transport protein; MFSD2a: main facilitator superfamily domain containing 2a; FABPpm: plasma membrane’s fatty acid binding protein.

In the placenta, FA are used for its growth and development or mobilized to the fetus mainly during mid and late pregnancy. Maternal TAG, by the action of placental lipases, are hydrolyzed to NEFA. Then, the placenta can uptake and move the NEFA across the membrane through a flip-flop mechanism or with the help of binding proteins and transporters (Figure 3) [95]. These include the placenta plasma membrane’s fatty acid binding protein (p-FABPpm), fatty acid translocase (FAT/CD36), and fatty acid transport proteins (FATP) [65]. Functionally, p-FABPpm is exclusively expressed in the apical membrane and exhibits a high affinity for LCPUFA, suggesting that this protein is involved in the preferential uptake of these PUFA. FAT/CD36 is a class B scavenger receptor protein, located both at the apical and basal membranes, and it is involved in angiogenesis, atherosclerosis, inflammation, and lipid metabolism. FATP are integral membrane proteins important for the cellular uptake of LCPUFA. The FATP family consists of at least six related members, of which five are expressed in the human placenta (FATP1-4, and 6; *SLC27A1-4,* and *6*). It is important to note that these FATP do not have specificity for a certain type of FA. Fatty acid binding proteins (FABP) are cytosolic proteins allowing for the trafficking of FA to sites within syncytiotrophoblast for esterification, β-oxidation, or transfer to the fetus. The human placenta expresses four different isoforms of FABP: FABP1, FABP3, FABP4, and FABP5 (*FABP1, 3, 4,* and *5*) [94,96]. In addition, it was recently established that the placenta expresses the major facilitator superfamily domain-containing protein 2 (MFSD2a) [97], which is a sodium-dependent lysophospholipid transporter known for its expression at the blood brain barrier. MFSD2a uptakes DHA in the form of lysophosphatidylcholine (LPC) [98]. Interestingly, the placental expression of MFSD2a is correlated with DHA levels in cord blood, suggesting its role in the maternal-fetal transfer of DHA [97]. 

In pregnant women, excessive fat mass increases the circulating levels of pro-inflammatory cytokines, IL-6, and tumor necrosis factor- α (TNF-α), among others [99], which further accentuates pregnancy-induced insulin resistance. These pro-inflammatory molecules impair, mainly during the second and third trimester of gestation, multiple metabolic pathways that result in higher maternal circulating levels of TAG, LDL-, and VLDL-cholesterol [60]. Moreover, it has been observed that obesity promotes a lipotoxic placental environment with macrophage infiltration and high levels of IL-1, TNF-α, IL-6, and oxidative stress, in addition to alterations in energy metabolism [21]. Cytokines regulate the expression, activity, and subcellular localization of placental FA transporters. In this regard, it has been shown that IL-6 stimulates trophoblast FA accumulation [100]. The effect of oxidative stress on the placental transport of FA is unknown, but it has been hypothesized that it could produce an increase in pro-inflammatory cytokines, thus indirectly regulating placental lipid metabolism (Figure 1) [101]. On the other hand, few studies have pointed out the role of maternal PUFA consumption on placental inflammation and oxidative stress. Both in human and rodent placenta, supplementation with *n*-6 PUFA does not seem to modify the inflammation that occurs at the term of pregnancy, although it slightly elevates the gene expression of pro-inflammatory cytokines such as TNF-α, IL6, and IL1-β. On the other hand, *n*-3 PUFA supplementation increases resolvin and protectin levels, which are derived from DHA and EPA and have anti-inflammatory properties [102,103]. Moreover, *n*-3 PUFA intake during pregnancy reduces placental oxidative damage due to an increase in antioxidant capacity in the placental labyrinth zone at the final third of rat pregnancy [104]. Similar findings have been reported in placental BeWo cells and in human placental explants [105]. Interestingly, in vitro models have shown that high levels of DHA (100 µM) enhance lipid peroxidation and DNA oxidative damage in placental cells and explants [105]. On the other hand, pregnant rats fed with a LA-rich diet showed decreased placental concentrations of IL-7 and IL-10, indicating that this *n*-6 PUFA has both pro-inflammatory and anti-inflammatory properties in the placenta [106]. DHA seems to have beneficial effects on fetal growth but no effects on placental tissue in rat models. On the other hand, maternal AA levels were positively correlated with placental lipid peroxidation and fetal growth restriction, mainly in female fetuses, indicating a sex-dependent effect [107]. It is interesting to note that there is evidence suggesting that fetal PUFA demands vary in a sex-dependent way. In pregnant sows, García-Contreras showed that the male fetus has higher ratios of MUFA to SFA and C18:1 to C18:0, indicating higher stearoyl-CoA desaturase activity in the liver [108]. Moreover, male fetuses had a higher *n*-6/*n*-3 ratio than females [108].

Experimental evidence in ovine models indicates that maternal obesity alters placental FA transport through changes in transporter levels rather than TAG [109]. On the other hand, studies in human placenta have found similar findings but have been inconsistent regarding the expression of different transporters and binding proteins. Hirschmugl et al. observed that maternal obesity significantly modified the expression of placental genes related to the transport and storage of neutral lipids (*ATGL, FATP1, FATP3, PLIN2, PPARG*, and *CGI-58*), resulting in a higher content of placental TAG [110]. Dubé et al. found an increase in mRNA levels and protein expression of FAT/CD36, while both FABP1 and FABP3 were found to be decreased [111]. Lager et al. showed that FATP2 expression was correlated with maternal BMI in contrast to FAT/CD36 and FATP4 [112]. Interestingly, Calabuig-Navarro et al. observed a higher esterification and storage of lipids, lower mitochondrial fatty acid oxidation, but elevated peroxisomal fatty acid oxidation in human placenta from mother with obesity compared to from normal-weight mothers [113]. The authors concluded that these changes suggest an adaptative mechanism that limits the transfer of FA to the fetus in case of maternal obesity [113].

Evidence from studies that have measured FA in erythrocytes from cord blood in women with gestational obesity indicates that the modifications in FA transporters and placental uptake are reflected by an altered FA profile in the fetal circulation characterized by decreased PUFA, both total *n*-6 and DHA (Figure 2) [82]. A recent study that looked at the placental transport of 13C-PUFA in pregnant women with obesity described a higher concentration of LA and DHA in the maternal plasma NEFA fraction of mothers with obesity. Nevertheless, the umbilical cord venous to maternal plasma ratio of 13C-LA was lower, whereas 13C-DHA tended to be lower in women with obesity compared to controls, suggesting a reduced maternal-fetal transfer of these FA [21]. However, Draycott et al. observed that fetal 13C-LA concentrations seem to be influenced by the total amount of FA in the maternal diet, more so than by the *n*-6 to *n*-3 ratio, concluding that a higher amount of total FA consumed would favor the placental transport of 13C-LA [114]. In addition, Shrestha et al. [14] observed that the exposure to LA increased the expression of the transporters FATP1, FATP4, and FABP5 and decreased that of FABP3 in a culture of cytotrophoblasts, indicating that these proteins could regulate the transport of this PUFA. On the other hand, studies on the placental transport of 13C-LA are controversial, and it is still unclear whether maternal-fetal transfer is affected by obesity. Although a higher LA uptake has been reported in isolated cytotrophoblasts from women with obesity, this effect is not maintained after the differentiation into syncytiotrophoblast [111]. On the other hand, a preliminary study of a placental metabolomic analysis of obese women showed an increase in PA concentration and a decrease in DHA, AA, and stearic acid (C18:0, SA) [115]. However, these results could be influenced by the presence of GDM. Placenta explants from obese women did not show any differences in the uptake of AA and DHA, but those from women with obesity and male fetus showed an increased uptake of OA [116].

In general, changes in the placental transfer of FA induced by maternal obesity could lead to alterations in different fetal tissue, such as the liver, adipose tissue, muscle, and brain. In humans, pre-pregnancy obesity is associated with increased oxidative stress in the newborn; a study in pregnant women showed a direct relationship between umbilical cord malondialdehyde levels, a biomarker of oxidative stress, and maternal BMI [117]. With regards to *n*-3 PUFA, it has been observed that, depending on experimental conditions, the administration of *n*-3 PUFA can have both pro and antioxidant effects [91,118]. Consistently, Shoji et al. showed that modest levels of DHA alleviate oxidative DNA damage, whereas high levels of DHA accelerate lipid peroxidation [118].

## 5. Impact of Maternal Obesity on PUFA Metabolism and Fetal Development

Maternal obesity alters the structure and function of the placenta, leading to modifications in the PUFA delivered to the fetus, producing changes in fetal development that can result in metabolic disturbances within multiple organs (Figure 1) [119,120]. Once FA are transferred to the fetal circulation, they are mobilized to tissue binding through α-fetoprotein [121]. In baboons, visceral fetal organs such as the liver, kidney, and lung have the capacity to synthesize AA from LA. In the heart, on the other hand, LA is more important for oxidation than for AA synthesis [122]. One study that analyzed fetal liver microsomes showed FADS1 and FADS2 activities in human fetal liver as early as 18 and 22 weeks of gestation, but these are lower than in other species, especially rodents [123,124]. Therefore, fetal organs have the capacity to produce DHA from ALA and AA from LA, but these capacities appear to be limited, suggesting that DHA and AA must also be obtained from the mother [125].

Although the gene expression of the enzymes required for de novo lipogenesis and fatty acid oxidation (FAO) have been reported in the fetal liver, it is considered that in this organ FA catabolism is suppressed in comparison to what happens during the postnatal period, as the major source of energy supply for the fetus comes from carbohydrates and amino acids rather than fat [126]. However, there is evidence showing a high rate of lipogenesis in slices from murine fetal liver compared to those from adult liver [5,127]. Measurements in murine and human tissues indicate that the activity of enzymes involved in FAO and the presence of different acylcarnitines have also been reported, indicating that these processes are functional in the fetal liver [128,129].

Studies performed in primates and mice have shown that HFD-induced maternal obesity has been associated with lipid accumulation in the fetal liver, suggesting that an abnormal supply of FA could alter the fetal hepatic FA metabolism, and this could be an early mechanism contributing to the origin of NAFLD [130,131]. It is interesting to note that non-human primate models do not develop obesity during pregnancy but show fat accumulation in the fetal liver, suggesting a dietary role in this process. Gene expression of de novo lipogenesis enzymes, such as fatty acid synthase (FAS) and sterol regulatory element-binding protein 1 (SREBP-1), is increased in the fetal liver of non-human primates fed with an obesogenic diet [126]. On the other hand, rats with gestational obesity induced by a diet rich in saturated fat exhibited a decreased gene expression of FAO enzymes [132]. Together with these changes, alterations in the plasma FA profile have been found showing reduced levels of *n*-6 DPA concomitantly with elevated plasma levels of LA and AA (Figure 2) [133]. Interestingly, the consumption of a HFD enriched with dietary *n*-3 PUFA, such as ALA, EPA and DHA in dams leads to a reduction in TAG accumulation in the fetal liver, suggesting that the maternal diet composition is fundamental in the prevention or worsening of this condition [134].

Maternal supplementation with *n*-3 PUFA during pregnancy decreases SREBP-1 protein expression and increases peroxisome proliferator-activated receptor alpha (PPAR-α), which promotes FAO in the offspring’s liver at 3 days of age [135]. Maternal obesity can also produce a mitochondrial dysfunction, evidenced by impairments in the fetal hepatic respiratory chain capacity, which could contribute to increased oxidative stress [132,136,137,138]. However, there is no evidence about how PUFA can regulate the ROS levels in fetal liver, which could be of interest in order to avoid early damage in the liver.

PUFA are stored in fetal adipose tissue, such that at the end of pregnancy the amounts of DHA and AA are several times higher in fetal than in maternal adipose tissue [139]. Moreover, LCPUFA act on preadipocytes, promoting adipogenesis due to transcriptional expression regulation of genes related to lipid metabolism, such as those encoding for the PPAR family [5]. In maternal obesity, evidence from animal and human studies supports the hypothesis that an imbalance of essential PUFA at early stages of pregnancy alters fetal adipose tissue development, favoring an abnormal lipid metabolism [140]. Similarly, the excess and imbalance of PUFA in fetal circulation induce alterations of skeletal muscle development, resulting in changes in its function and metabolism that predispose the child to future metabolic disorders. A study conducted in sheep suggests that maternal obesity induced by HFD generates modifications in fetal myogenesis and in various signaling cascades, such as the insulin signaling pathway, adipogenesis, and oxidative stress pathways [141]. Another group found a reduced muscle fiber density that could be associated with an increase in adipogenesis (diversion from myogenesis to adipogenesis) and lipid accumulation in fetuses of obese pregnant sheep [142]. Interestingly, studies in cell lines have shown that EPA and DHA reduce myogenesis and increase adipogenesis in myotube formation [143].

PUFA are central in fetal brain development. In this regard, DHA is particularly important because it regulates cell survival, neuroinflammation, and neurogenesis and participates in signal transduction and blood-brain barrier (BBB) permeability [13,144]. AA is crucial for several functions in the brain, such as neuronal firing, signaling, and long-term potentiation. Moreover, AA contributes to the maintenance of hippocampal plasticity in part because it activates the PPARγ [145].

The absorption of esterified lipids through the BBB is essential for the accumulation of DHA, in which its dissociation from albumin lysophospholipids or the release of circulating lipoproteins by lipases of brain endothelium are required to cross to the luminal plasma membrane of the BBB by passive diffusion or facilitated transport [144]. FA bind FABP for trafficking within the cell or are trapped by the activation to acyl-CoA catalyzed by the activity of FATP [13,144]. FABP7 levels correlate with neuronal differentiation, whereas FABP5 influences endothelial cells within the brain. Recently, it has been proposed that brain uptake of DHA occurs mostly through MFSD2a, which uptakes lysophospholipids at the BBB [144,146], although this is still controversial. 

Hypothalamic inflammation induced by HFD has been shown to deregulate energy homeostasis, leading to insulin resistance, glucose intolerance, and obesity [147,148]. There are several possible mechanisms that can induce hypothalamic inflammation through the activation of different intracellular processes in the hypothalamic glial cells, including oxidative stress, endoplasmic reticulum stress, RNA stress, autophagy defects, or the activation of toll-type receptors (TLRs) and cytokines. Most of these intracellular processes converge in the activation of the N-terminal kinase c-Jun (JNK) and the IκB kinase-nuclear factor kappa B (IKK/NF-κB) [148,149,150,151]. It has been observed in rats that maternal obesity induces an upregulation of several members of the toll-like receptor 4 signaling cascade and the subsequent activation of inflammatory pathways in the hypothalamus [152].

## 6. Impact of Maternal Obesity on PUFA Metabolism during Breastfeeding and Neonatal Development

Clinical trials have shown that the composition of breast milk appears to be determined through adaptations that occur during pregnancy with the aim of supporting the newborn according to the gestational age [153]. During pregnancy, prolactin in concert with glucocorticoids and insulin are the main inductors of breast development and differentiation. Twenty-four hours after birth, the drop of progesterone along with glucocorticoids induce cellular and molecular changes in the mammary epithelium, resulting in modifications of key pathways of carbohydrate and lipid metabolism, in addition to increasing protein synthesis and milk production [154]. In turn, prolactin induces the expression of lipogenic genes involved in de novo FA lipogenesis and β-oxidation in the mammary gland both in humans and mice [155,156].

Breast milk composition can be influenced by maternal age, weight, diet, and health condition [157]. Moreover, it varies throughout the lactation period. First, milk corresponds to colostrum produced until the 5th day of lactation, then transition milk, until the 14th day, and finally mature milk [157]. Colostrum is characterized by a high content of protein (23 g/L) and immunological factors. This milk is rich in *n*-6 and *n*-3 PUFA, specifically LA and ALA, and is the initial source of LCPUFA to breastfed newborns [158]. LCPUFA composition of mature human milk in lactating women from Europe, Africa, Asia, and South America shows that the major LCPUFA in human milk are AA, DGLA, and eicosadienoic acid (C20:2*n*-6, EA) from *n*-6 series in addition to DHA and DPA from *n*-3 series [159]. Overall, the concentration of FA in breast milk increases from the first to the fourth month of lactation and then remains stable until the 6th month. However, *n*-3 LCPUFA and DHA decrease from the 4th month of lactation, resulting in a high *n*-6 to *n*-3 LCPUFA ratio [19]. Although DHA content in breast milk varies in relation to the mother’s DHA intake, a global average of DHA in breast milk has been estimated at 0.32 ± 0.22% [160]. Interestingly, DHA supplementation was shown to slightly reduce *n*-6 LCPUFA levels in breast milk, which reduces the *n*-6: *n*-3 ratio, suggesting that DHA could modify the transport or metabolism of *n*-6 LCPUFA [161].

Maternal obesity impacts breastfeeding because the excess of adipose mass affects hormonal regulation and alters the composition of breast milk (Figure 1). In this regard, obese women had reduced levels of prolactin compared to women with normal weight [162]. In addition, insulin resistance and impaired insulin levels, which are closely related to obesity, delay the onset of lactogenesis and alter milk production [163]. These effects are mediated, in part, by an abnormal de novo lipogenesis related to an increased AMP-activated protein kinase (AMPK) activity, the inhibition of acetyl-CoA carboxylase, and a decreased PUFA synthesis in the mammary gland [164]. This evidence explains why women with obesity breastfeed for a shorter duration and introduce complementary food earlier than women with normal weight [165]. Moreover, a study conducted by Lima et al. in pregnant rats divided into two groups, one control and one fed with a HFD, showed that maternal lipids are reflected in milk composition, and a HFD leads to hypercholesterolemia and visceral fat accumulation in the offspring [166]. Maternal obesity is also associated with the alteration of immunological factor concentrations in human milk, such as C-reactive protein (CRP), leptin, IL-6, insulin, TNF-α, ghrelin, adiponectin, and obestatin. Moreover, excessive weight and obesity appear to have a clear influence on the immunological properties of human milk [167]. The alterations of bioactive properties in the human milk of obese mothers could increase the incidence of obesity, insulin resistance, type 2 diabetes, and other adverse metabolic outcomes in the offspring [168]. Despite this, there is not enough evidence to suggest that these changes should preclude infant’s breastfeeding, because the benefits of maternal milk are well established [169].

In the colostrum of women with obesity, higher levels of LA and AA and lower levels of ALA and DHA have been observed compared to normal-weight mothers [10]. The FA profile of mature breast milk from mothers with obesity is characterized by: (i) increased SFA levels, mainly PA; (ii) decreased MUFA, such as OA; (iii) lower *n*-3 PUFA, including ALA, DPA, EPA, and DHA; (iv) higher *n*-6 PUFA, DGLA, and adrenic acid (C22:4*n*-6, ADA); and (v) elevated *n*-6 to *n*-3 ratio [168,169,170,171,172,173,174,175]. Similarly, the breast milk of Japanese monkeys fed with a HFD exhibited lower levels of EPA and DHA, along with a higher *n*-6 to *n*-3 ratio than those fed with a control diet [176]. Interestingly, there are other types of lipids with anti-inflammatory and anti-diabetics properties in human breast milk, like palmitic acid and hydroxystearic acids, which were found to be lower in the milk of obese mothers compared to normal-weight mothers [177]. In general, these modifications can be attributed to differences in the mobilization of endogenous FA stores, synthesis in the maternal liver and breast tissue, or dietary differences [174]. In this sense, a maternal diet with low levels of essential PUFA results in a decrease of PUFA levels in breast milk (Figure 2) [168].

The content of PUFA from *n*-3 and *n*-6 series in breast milk has been associated with modifications in weight and fat distribution in the infant. It has been established that the colostrum content of AA and DHA is inversely correlated with infant BMI at 6 months of age, whereas the *n*-6 to *n*-3 ratio is positively associated with BMI. Moreover, in infants of overweight mothers, DHA and *n*-3 LCPUFA levels in colostrum were positively associated with cognitive scores, while the *n*-6 to *n*-3 ratio was inversely associated with it. Moreover, SFA, as well as the ratio of unsaturated to SFA in mature breast milk at 3 months, are correlated with children’s weight gain at 13 months [169]. In turn, a positive association between ALA levels in mature milk with cognition has been observed in infants born to mothers with obesity [170].

Several studies have shown that the type of dietary FA consumed by dams during pregnancy and/or lactation can have beneficial or adverse consequences on the health of their offspring (Figure 2). The maternal consumption of diets rich in trans FA and SFA can lead to impairments in the metabolism and development of the offspring, basically by TLR4 activation and disturbances in glucose and lipid homeostasis. However, the majority of the studies evaluating the early exposition to PUFA, in particular *n*-3 PUFA, have shown benefits in the offspring development and epigenetic regulation, which seem to play a role in the prevention of obesity, insulin resistance, and the risk of developing cardiovascular diseases later in life. In relation to MUFA, limited evidence indicates that maternal intake can stimulate thermogenic capacity and change liver metabolism, favoring the offspring’s health [178]. In animal models and clinical trials based on *n*-3 PUFA supplementation during pregnancy and lactation, researchers have demonstrated the importance of these FA during breastfeeding. However, the results of these studies are heterogenous. A study in mice with maternal EPA + DHA-rich fish oil supplementation potentiated the development of fetal brown adipose tissue, a crucial regulator of energy expenditure that reduces the susceptibility to develop obesity [179,180]. On the other hand, male and female rat offspring born to dams supplemented with a diet enriched with EPA and DHA exhibited large subcutaneous fat depots without effect on the expression of adipogenic or lipogenic genes in adipose tissue at early postnatal life [178,181].

In women with maternal obesity, Rouille et al. showed a positive relationship between the maternal ratio of *n*-3 PUFA to total PUFA intake and fetal growth in overweight pregnant women [182]. Likewise, a randomized controlled trial involving 556 pregnant women observed that women with obesity supplemented from mid-pregnancy until delivery with 2 g/d of marine *n*-3 PUFA containing 800 mg of DHA plus 1200 mg of EPA had an attenuated increment in plasma *n*-3 concentrations and a lower reduction of *n*-6 to *n*-3 ratio compared to women with normal weight [183]. The serum concentration of *n*-3 PUFA was positively associated with the sum of skinfold at one year of age in women’s offspring supplemented with DHA during pregnancy and lactation [74]. Likewise, DHA and *n*-3 PUFA of early breast milk were positively related to the proportion of subcutaneous and preperitoneal fat (subcutaneous/preperitoneal) assessed by ultrasound in offspring at 6 weeks postpartum [184]. Similar observations were made in a Spanish cohort in which the authors found that *n*-3 PUFA were positively related to weight and the percentage of lean mass in the infants [5], but no associations were found in infants of mothers with overweight or obesity [5].

There is evidence indicating that supplementation with *n*-3 PUFA can improve some neurodevelopment parameters. In a study in which the school menu was modified to offer fish twice a week to 8–10 year-old healthy children, the authors report a significantly improved school performance and reading comprehension, in addition to increases in EPA and DHA levels [185]. 

Similarly, Richardson et al. conducted a randomized controlled trial in 5–12 year-old children with developmental coordination disorders and observed that daily supplementation with *n*-3 PUFA (558 mg of EPA and 174 mg of DHA), *n*-6 PUFA (60 mg of γ-linoleic acid), and vitamin E (9.6 mg of α-tocopherol) resulted in improvements in reading, spelling, and behavioral scores [186]. These data suggest that a supplementation with *n*-3 PUFA could improve some of the parameters that cause neurodevelopmental abnormalities associated with gestational obesity.

## 7. Conclusions

Maternal obesity and excessive GWG are strongly associated with changes in the FA profile of maternal and fetal circulation during pregnancy and with breast milk composition during lactation. Moreover, the pro-inflammatory status induced by obesity alters placental FA transporters, resulting in an imbalance in the transport of nutrients, including FA. Additionally, maternal diets and hormonal and metabolic modifications affect breastfeeding and breast milk composition. In general, obesity leads to modifications in PUFA levels in breast milk and fetal circulation with lower *n*-3 PUFA (mainly EPA and DHA) and higher *n*-6 PUFA (mainly LA and AA) resulting in a high *n*-6 to *n*-3 ratio. Early nutrition patterns in fetuses and neonates can influence adiposity, in addition to the risk of developing metabolic and behavioral disorders, and these associations can persist throughout the children’s whole life.

## Figures and Tables

**Table 1:** Pregnant and lactating women’s dietary recommendations.

Fat Intake	FAO/WHO [66]	Dietary Reference Intake [70,71]
Fat	20–35% E	20–35% E
MUFA	Difference	--
SFA	Max 10% E	<10% E
PUFA	6–10% E	--
*n*-6 PUFA	2.5–9% E	5–10% E
*n*-3 PUFA	0.5–2% E	0.6–1.2% E
DHA	200 mg/d	1.4 g/d ALA
EPA + DHA	300 mg/d	--
AA	800 mg/d	13 g/d LA
Trans fatty acids	<1% E	--

% E (% of total energy); MUFA, monounsaturated fatty acids; SFA, Saturated fatty acids; PUFA, polyunsaturated fatty acids; ALA, α–linolenic acid; AA, arachidonic acid; DHA, docosahexaenoic acid; EPA, eicosapentaenoic acid; LA, linoleic acid.

## Abbreviations

AA	arachidonic acid
ADA	adrenic acid
AL	α–linolenic acid
AMP	AMP-activated protein kinase
ASD	autism spectrum disorder
ATG	adipose triglyceride lipase
BBB	blood-brain barrier
BMI	body mass index
CGI-58	comparative gene identification-58
CRP	C-reactive protein
DGLA	dihomo-γ-linolenic acid
DH	docosahexaenoic acid
DPA	docosapentaenoic acid
EARNEST	Early Nutrition Project
ELOVL	elongation of very long chain fatty acids protein
EA EPA	eicosadienoic acid eicosapentaenoic acid
FABP FADS	fatty acid binding protein fatty acid desaturase
FAO	fatty acid oxidation
FAS	fatty acid synthase
FA	fatty acid
FAT/CD36	fatty acid translocase
FATP	fatty acid transport protein
GDM	gestational diabetes mellitus
GWG:	gestational weight gain
HFD IKK/NF-κB	high fat diet IκB kinase-nuclear factor kappa B
IκB	kinase-nuclear factor kappa B
IL	interleukin
JN	kinase c-Jun
LA	linoleic acid
LCPUFA	long chain polyunsaturated fatty acid
MFSD2a	main facilitator superfamily domain containing 2a
MUFA	monounsaturated fatty acid
NAFLD	non-alcoholic fatty liver disease
NA	nervonic acid
NEFA	non-esterified fatty acid
OA	oleic acid
PA	palmitic acid
PeriLip	Perinatal Lipid Nutrition Project
p-FABPpm	placenta plasma membrane fatty acid binding protein
PLIN2	perilipin 2
POA	palmitoleic acid
PPAR-γ	peroxisome proliferator-activated receptor gamma
PPAR-α	peroxisome proliferator-activated receptor alpha
ROS SA SFA	reactive oxidative species stearic acid saturated fatty acid
SREBP-1 TAG	sterol regulatory element-binding protein 1 triacylglycerols
TLRs	toll-type receptors
TNF-α	tumor necrosis factor-alpha
VLDL	very low-density lipoprotein
